# Social Disconnectedness in Individuals with Cardiovascular Disease: Associations with Health Literacy and Treatment Burden

**DOI:** 10.1007/s12529-024-10263-9

**Published:** 2024-03-13

**Authors:** Julie Christiansen, Mathias Lasgaard, Susanne S. Pedersen, Marie Hauge Pedersen, Karina Friis

**Affiliations:** 1 Department of Psychology, University of Southern Denmark, Odense, Denmark; 2 DEFACTUM – Public Health Research, Central Denmark Region, Aarhus, Denmark; 3 Department of Cardiology, Odense University Hospital, Odense, Denmark; 4 Department of Public Health, Aarhus University, Aarhus, Denmark

**Keywords:** Loneliness, Social isolation, Health literacy, Treatment burden, Cardiovascular disease

## Abstract

**Background:**

Knowledge is limited on associations between social disconnectedness (i.e. loneliness and social isolation), health literacy and perceived treatment burden in individuals with cardiovascular disease (CVD). However, understanding these associations may be important for clinical practice.

**Methods:**

This study used cross-sectional self-reported data from the 2017 Danish health and morbidity survey entitled ‘How are you?’, investigating the associations of loneliness and social isolation with low health literacy and high treatment burden in individuals with CVD (*n* = 2521; mean age = 65.7 years).

**Results:**

Logistic regression analysis showed that loneliness and social isolation were associated with low health literacy in terms of difficulties in ‘understanding health information’ (loneliness: adjusted odds ratio (*AOR)* = 1.32, 95% confidence intervals (*CI)* [1.16, 1.50]; social isolation: *AOR* = 1.47, 95% *CI* [1.24, 1.73]) and ‘engaging with healthcare providers’ (loneliness*: AOR* = 1.53, 95% *CI* [1.37, 1.70]; social isolation: *AOR* = 1.21, 95% *CI* [1.06, 1.40]) and associated with high treatment burden (loneliness: *AOR* = 1.49, 95% *CI* [1.35, 1.65]; social isolation: *AOR* = 1.20, 95% *CI* [1.06, 1.37]).

**Conclusions:**

Our findings show that loneliness and social isolation coexisted with low health literacy and high treatment burden in individuals with CVD. These findings are critical as socially disconnected individuals experience more health issues. Low health literacy and a high treatment burden may potentially exacerbate these issues.

## Introduction

Social disconnectedness (e.g. loneliness and social isolation) is associated with increased risk of morbidity and mortality [1–3]. These associations are believed to be comprised of behavioural, psychological, and physiological pathways [4–6]. This implies that social disconnectedness influences health behaviours, triggers conditions like stress and depression, and impacts cardiovascular, neuroendocrine, immune, and inflammatory mechanisms. This complex interplay gives rise to detrimental conditions that increase the likelihood of incident disease. In particular, loneliness, a subjective, unpleasant emotional state resulting from a discrepancy between desired and achieved levels of social contact [7], and social isolation, the absence of social contacts and social relationships [8], are associated with increased risk of cardiovascular disease (CVD) [1–3].

Among individuals with CVD, social isolation has been associated with increased all-cause mortality [9, 10], while both loneliness and social isolation have been found to increase the use of healthcare services (i.e. more days hospitalised and more hospital readmissions) [11, 12]. As such, loneliness and social isolation do not merely increase the risk of incident CVD, rather, they seem to aggravate the course of disease. Consequently, the American Heart Association (AHA) has issued a scientific statement emphasising the importance of understanding the independent effects of social isolation and loneliness on cardiovascular and brain health, highlighting the need to intervene to reduce social isolation and loneliness to help advance health equity [13].

Despite the extant knowledge, gaps persist in our understanding of the adverse effects of loneliness and social isolation on the course of disease in individuals with CVD. Especially, our understanding of the underlying mechanisms through which social disconnectedness influences disease trajectory remains poorly elucidated. While the aforementioned pathways may provide partial insights, an investigation into the role of specific factors pertinent to individuals facing CVD may be warranted to obtain a more comprehensive understanding. Such specific factors might include health literacy, defined as *the degree to which people are able to access, understand and communicate health information, and engage with the demands of different health contexts in order to promote and maintain good health* [14], and perceived treatment burden, *the perceived workload of healthcare and its impact on patient functioning and well-being* [15]. As such, low health literacy and a high perceived treatment burden are more prevalent among individuals with CVD, than individuals with other chronic diseases [16, 17]. Furthermore, in individuals with CVD, low health literacy is associated with increased risk of mortality [18] as well as higher healthcare utilisation [18, 19]. Similarly, a high perceived treatment burden may pose a barrier to maintaining good health [20]. For instance, individuals with heart failure described that treatment burden reduced their capacity to follow treatment plans [21] and induced poor adherence [21, 22]. Taken together, low health literacy and a high perceived treatment burden might be considered critical to self-care in patients with CVD, potentially impacting prognostic outcomes, mortality, and healthcare utilisation.

Qualitative studies suggest that individuals with chronic diseases often draw on the health literacy competencies of their social network [23], underscoring the role of social connection for health literacy. Especially, interpersonal connections are important serving instrumental in rectifying and shaping health behaviours to prevent or mitigate health issues. This process includes fostering habits like physical activity and healthy dietary habits, as well as supporting the management of medications and engagement with healthcare [24]. Hence, social disconnectedness might limit opportunities for individuals to actively participate in and acquire vital health literacy skills, particularly impacting the adaptation and modification of health behaviours. Similarly, qualitative research has found that social isolation diminishes the capacity to manage treatment workload, increasing the perceived treatment burden [25]. The absence of social support, such as lack of family assistance, has been identified as one of several ascendants to perceived treatment burden [26]. Additionally, poor mental health is associated with an elevated likelihood of experiencing a high perceived treatment burden [26]. This aligns with the cumulative complexity model [27], positing that the utilisation of healthcare services and the practice of self-care demand adequate capacity, including social support and social resources, to bear the load of treatment responsibilities [28]. Consequently, factors linked to loneliness and social isolation, such as inadequate social resources and poor mental health including perceived stress and negative affectivity, may together contribute to a heightened treatment burden.

In sum, socially disconnected individuals may lack essential health literacy skills or experience higher perceived treatment burden than socially connected individuals. This is critical as socially disconnected individuals already experience more health issues, and low health literacy and a high perceived treatment burden may accelerate or aggravate such issues. Nonetheless, a paucity of research exists on the associations of loneliness and social isolation with health literacy and treatment burden in individuals with CVD. Knowledge of these associations is crucial, potentially enhancing our understanding of the mechanisms linking loneliness and social isolation with prognosis in individuals with CVD.

In the present paper, we examine the cross-sectional associations of loneliness and social isolation with two dimensions of health literacy and high perceived treatment burden in individuals with CVD. We hypothesise that loneliness and social isolation are associated with *low* health literacy and a *high* perceived treatment burden in individuals with CVD.

## Methods

This cross-sectional study used data from the Central Denmark Region 2017 health and morbidity survey ‘How are you?’ [29], which forms part of the Danish National Health Survey — a large representative population-based survey conducted in the Danish population [30]. The survey was based on a random sample of 52,000 individuals (16 + years) drawn from the Danish Civil Registration System using their personal civil registry number. A total of 28,627 respondents aged 25 + years completed the questionnaire (response rate: 63,6%); among these, 2521 respondents self-reported having CVD, i.e. acute myocardial infarction, angina pectoris, or stroke, comprising the present sample. Respondents were asked if they had the specific condition or had the condition previously [31]. The present study includes respondents who reported that they currently had one or more of the CVDs as well as those who had a history of these diseases.

### Measures

*Loneliness* was assessed using the Three-Item Loneliness Scale (T-ILS; [32]). The three questions of the T-ILS (*How often do you feel isolated from others? How often do you feel you lack companionship? How often do you feel left out?*) are rated on a three-point Likert scale (hardly ever, sometimes, often). The sum of the items (ranging from 3 to 9) provides a global loneliness measure, with a higher score indicating greater loneliness. The summed score was used as an indicator of loneliness. If more than one item was missing on the scale, cases were excluded (*n* = 115). *Social isolation* was measured using an index based on questions about social contact inspired by similar indexes proposed by Steptoe et al. [33] and Valtorta et al. [3]. The following indicators were included: (1) living alone, (2) less than monthly contact with family with whom one does not live, (3) less than monthly contact with friends, (4) less than monthly contact with colleagues/fellow students outside the workplace or school, (5) less than monthly contact with neighbours or the local community, and (6) less than monthly participation in community activities, religious gatherings, or voluntary work. The summed score (ranging from 0 to 6) was used as an indicator of social isolation, with higher scores reflecting greater social isolation. If more than one item was missing from the index, cases were excluded (*n* = 123). *Health literacy* was measured using two subscales from the comprehensive Health Literacy Questionnaire [34, 35], i.e. ‘understanding health information (well enough to know what to do)’ and ‘(ability to actively) engage with healthcare providers’. Each subscale consists of five items rated on a four-point Likert scale from ‘very difficult’ to ‘very easy’. The scale sums were calculated as the mean of the five-item scores and then standardised to range between 1 (lowest ability) and 4 (highest ability) to ensure consistency with the response format [35]. Each scale was coded into a binary variable corresponding to a maximum score of two to classify respondents who found it very difficult or difficult to ‘understand health information well enough to know what to do’ or to ‘actively engage with healthcare providers’ [19]. If more than two items were missing on either scale, cases were excluded (‘understand health information’: *n* = 140; ‘actively engage with healthcare providers’: *n* = 128). *Treatment burden* was measured using the Multimorbidity Treatment Burden Questionnaire (MTBQ; [17, 36]. The MTBQ comprises ten items, presenting different aspects of treatment burden, including the burden of managing one’s health, the burden related to medication, the burden of coordinating and travelling to attend healthcare appointments, and the burden of being dependent on others. All items are rated as follows: not difficult/does not apply to me (0), a little difficult (1), quite difficult (2), very difficult (3), and extremely difficult (4). To compute a global score of treatment burden, the average score was calculated from the completed items and multiplied by 25 to yield a sum score between 0 and 100. Respondents scoring 22 or above were classified as having a high treatment burden [36]. If more than five items were missing on the scale, cases were excluded (*n* = 129).

*Sociodemographic factors* included *sex* (binary), *age* (continuous), *educational attainment*, and *country of origin* (Danish/Non-Danish origin). *Sex*, *age*, and *country of origin* were obtained from Danish register data with no missing data, whereas *educational attainment* was based on self-reported information and classified using the Danish version of the International Standard Classification of Education (low educational level [0–10 years], medium educational level [11–14 years], and high educational level [≥ 15 years]) [37].

*Additional chronic disease* comprised self-reported information about asthma, allergy, diabetes, hypertension, chronic bronchitis/chronic obstructive pulmonary disease (COPD), arthritis (osteoarthritis and rheumatoid arthritis), osteoporosis, cataract, cancer, migraine/recurrent headaches, slipped discs/back pain, tinnitus, and mental illness. Respondents were classified as having a specific chronic disease if they had the disease at the time of the survey.

### Data Analysis

Logistic regression analyses were conducted to examine associations of loneliness and social isolation with low health literacy and high treatment burden. Analyses were conducted separately for ‘understanding health information’, ‘engaging with healthcare providers’, and high treatment burden. Two models were examined; first, a model in which loneliness and social isolation were mutually adjusted for; this was followed by a model also adjusting for sociodemographic factors (sex, age, educational attainment, and country of origin) and each additional chronic disease (i.e. asthma, allergy, diabetes, hypertension, COPD, arthritis, osteoporosis, cataract, cancer, migraine/recurrent headaches, slipped discs/back pain, tinnitus, and mental illness). In addition, the Wald (*X*^*2*^) test was performed to test for equality of the estimates of loneliness and social isolation in all fully adjusted models.

Analyses of high treatment burden included only respondents reporting that they were in active treatment (receiving treatment, taking medication, undergoing rehabilitation, or attending regular check-ups) (*n* = 2162).

To enhance the representativeness of the study sample, weights were applied in all analyses to account for potential differences in selection probabilities and response rates. Weights were constructed by Statistics Denmark using a model-based calibration approach [38] taking into account different sampling probabilities and differential non-response [30]. The information used to compute the weights included sex, age, municipality of residence, highest completed level of education, ethnic background, hospitalisation, and occupational status.

## Results

Sample characteristics are presented in Table 1. Among the entire sample, 41% were female and the mean age was 66 years. Mean levels of loneliness and social isolation were 4.2 and 1.9, respectively. Of the sample, 11% found it difficult or very difficult to ‘understand health information’, whereas 12% found it difficult or very difficult to ‘engage with healthcare providers’. Lastly, 20% of the respondents in active treatment were categorised as having a high perceived treatment burden.

**Table 1:** Sample characteristics

	Sample (*n* = 2,521)
	*n (*%^a^)
**Sex**	
Male	1,507 (59.4)
Female	1,014 (40.6)
**Age** *(mean, SD)*	65.71 (14.5)
**Educational attainment**	
Low (1–10 years)	566 (26.1)
Medium (11–14 years)	1,438 (53.3)
High (≥ 15 years)	436 (17.6)
**Country of origin**	
Danish	2,440 (93.8)
Non-Danish	81 (6.2)
**Additional chronic disease**	
Asthma	216 (9.2)
Allergy	404 (16.6)
Diabetes	388 (15.9)
Hypertension	1165 (45.3)
COPD	318 (13.0)
Osteoarthritis	992 (39.5)
Rheumatoid arthritis	361 (16.0)
Cataract	300 (12.4)
Osteoporosis	225 (9.7)
Cancer	143 (5.4)
Migraine/recurrent headaches	379 (16.0)
Slipped discs’/back pain	603 (24.5)
Tinnitus	570 (21.9)
Mental illness	327 (14.8)
**Social disconnectedness**	
Loneliness *(mean, SD)*	4.24 (1.6)
Social isolation *(mean, SD)*	1.87 (1.4)
**Low health literacy**	
Difficulties in understanding health information	215 (10.8)
Difficulties in engaging with healthcare providers	248 (11.7)
**High treatment burden**^b^	338 (19.7)

*SD* standard deviation, *COPD* chronic obstructive pulmonary disease

^a^All percentages and means are weighted based on register data to represent the population of the Central Denmark Region, 2017

^b^Individuals not in treatment were excluded prior to analysis (*n* = 359)

In the mutually adjusted analyses, loneliness and social isolation were associated with higher odds of difficulties in ‘understanding health information’ (loneliness: odds ratio (*OR*) = 1.30, 95% *CI* [1.17, 1.44]; social isolation: *OR* = 1.63*,* 95% *CI* [1.40, 1.90]; Table 2). Likewise, both loneliness and social isolation were associated with higher odds of difficulties in ‘engaging with healthcare providers’ (loneliness: *OR* = 1.52*,* 95% *CI* [1.38, 1.67]; social isolation: *OR* = 1.28, 95% *CI* [1.12, 1.47]; Table 2). Adjusted for sociodemographic factors and additional chronic disease, loneliness and social isolation remained associated with higher odds of difficulties in ‘understanding health information’ (loneliness: adjusted odds ratio (AOR) = 1.32*,* 95% *CI* [1.16, 1.50]; social isolation: *AOR* = 1.47, 95% *CI* [1.24, 1.73]; Table 3) and ‘engaging with healthcare providers’ (loneliness*: AOR* = 1.53, 95% *CI* [1.37, 1.70]; social isolation: *AOR* = 1.21, 95% *CI* [1.06, 1.40]; Table 3). Subsequent Wald tests demonstrated a stronger association of loneliness than of social isolation with difficulties in ‘engaging with healthcare providers’ (Table 3).

**Table 2:** The associations of loneliness and social isolation with low health literacy and high treatment burden in mutually adjusted logistic regression analyses

	**Difficulties in understanding health information**^a^	**Difficulties engaging with healthcare providers**^b^	**High treatment burden**^c^
	OR (95% CI)	OR (95% CI)	OR (95% CI)
Loneliness	1.30* (1.17–1.44)	1.52* (1.38–1.67)	1.57* (1.43–1.72)
Social isolation	1.63* (1.40–1.90)	1.28* (1.12–1.47)	1.27* (1.12–1.44)
Wald (*X*^*2*^)	3.96*	2.81	5.22*

*OR* odds ratio, *CI* confidence intervals

*p < 0.05

^a^*n* = 2304

^b^*n* = 2311

^c^*n* = 1933

In the mutually adjusted analyses, loneliness and social isolation were associated with higher odds of a high perceived treatment burden (loneliness: *OR* = 1.57, 95% *CI* [1.43, 1.72]; social isolation: *OR* = 1.27, 95% *CI* [1.12, 1.44]; Table 2). When also adjusting for sociodemographic factors and additional chronic disease, loneliness and social isolation remained associated with higher odds of a high perceived treatment burden (loneliness: *AOR* = 1.49, 95% *CI* [1.35, 1.65]; social isolation: *AOR* = 1.20, 95% *CI* [1.06, 1.37]; Table 3). Subsequent Wald tests demonstrated a stronger association of loneliness than of social isolation with high perceived treatment burden (Table 3).

**Table 3:** The associations of loneliness and social isolation with low health literacy and high treatment burden in the fully adjusted logistic regression analyses

	**Difficulties in understanding health information**^a^	**Difficulties engaging with healthcare providers**^b^	**High treatment burden**^c^
	AOR (95% CI)	AOR (95% CI)	AOR (95% CI)
Loneliness	1.32* (1.16–1.50)	1.53* (1.37–1.70)	1.49* (1.35–1.65)
Social isolation	1.47* (1.24–1.73)	1.21* (1.06–1.40)	1.20* (1.06–1.37)
Wald (*X*^*2*^)	0.63	4.68*	4.78*

Adjusted for sex, age, educational attainment, country of origin and each additional chronic disease (asthma, allergy, diabetes, hypertension, chronic bronchitis/chronic obstructive pulmonary disease, arthritis (osteoarthritis and rheumatoid arthritis), osteoporosis, cataract, cancer, migraine/recurrent headaches, slipped discs/back pain, tinnitus and mental illness). Wald (*X*^*2*^) test performed to test for equality (e.g. between the estimates of loneliness and social isolation)

*AOR* adjusted odds ratio, *CI* confidence intervals

*p =  < 0.05

^a^*n* = 2279

^b^*n* = 2285

^c^*n* = 1933

## Discussion

The present findings contribute to the mounting body of literature on the associations of social disconnectedness with health and health-related outcomes. The main finding of the present study was that higher levels of loneliness and social isolation were associated with higher odds of reporting low health literacy, which applied to both difficulties in ‘understanding health information’ and difficulties in actively ‘engaging with healthcare providers’. Moreover, loneliness and social isolation were associated with higher odds of a high perceived treatment burden in individuals with CVD. Findings also indicated that the associations of loneliness with ‘engaging with healthcare providers’ and a high perceived treatment burden were more robust than the associations of social isolation with ‘engaging with healthcare providers’ and a high perceived treatment burden.

Despite the seeming relevance of investigating social disconnectedness in conjunction with health literacy and treatment burden in individuals with CVD, the findings from the present study are novel. Even so, the present findings are consistent with qualitative research showing that individuals with chronic disease may experience social disconnectedness together with low health literacy and a high perceived treatment burden [23, 25, 39]. Similarly, the findings are in line with studies showing that loneliness is associated with low health literacy among adolescents [40] and older adults [41, 42] and mirror findings that social support and a large social network are linked to better self-management skills and self-care behaviour in individuals with heart failure [43].

In line with evidence of loneliness and social isolation as risk factors for health and health-related outcomes [1–3], our findings may reflect that social disconnectedness increases the risk of low health literacy and a high perceived treatment burden. As such, social disconnectedness may increase the risk of low health literacy in individuals with CVD, i.e. a perceived or actual lack of social connections creates a shortage of essential skills otherwise shared among members within a group or between caregivers. Even though health literacy is often thought of as an individual attribute, individuals are thought to share their health literacy skills [23]. Thus, health literacy skills may be distributed through one’s social network, whereby the network comes to support its members in managing their disease, communicate with health professionals, and make decisions about their healthcare [23, 39]. Likewise, social factors, such as lack of support or assistance, have been suggested as one of several antecedents that may lead to treatment burden in individuals with chronic disease [26].

The pathways by which social disconnectedness impacts the onset of disease have been, to a certain extent, explored. However, the understanding of the mechanisms influencing the progression of disease in individuals with chronic conditions, such as CVD, remains relatively scarce. Low health literacy skills and a high perceived treatment burden might constitute plausible mechanisms through which loneliness and social isolation influence the trajectory of the disease course. This line of reasoning suggests a relationship wherein disconnectedness may contribute to the development of low health literacy skills and a high perceived treatment burden, subsequently culminating in adverse disease outcomes. Due to the present study’s cross-sectional nature, it is not possible to determine directionality or temporality. Our findings could therefore also reflect that low health literacy and a high perceived treatment burden give rise to feelings of loneliness or increase the risk of social isolation. For instance, some individuals with low health literacy may feel ashamed about this limitation [44]—especially when dealing with a chronic or severe disease requiring comprehensive disease management. Such feelings of shame may make individuals with low health literacy unwilling or unable to make use of social resources [41], potentially leading to social withdrawal. In a similar way, treatment burden may lead to disruptions in a person’s relational capacity [45], due to issues with navigating complex treatment regimens or by posing relationship strains due to time or financial resources spent on treatment [45, 46]. Future prospective studies are required to explore the relationships between social disconnectedness, health literacy skills, and high perceived treatment burden, aiming to establish the directionality of these associations. Similarly, further investigations are needed to delineate whether and to what extent the coexistence of social disconnectedness with low health literacy skills and high perceived treatment burden may impact the progression of disease in individuals with CVD.

### Clinical Implications

Even though the present study cannot determine directionality, our findings have important clinical implications. The findings suggest that loneliness and social isolation coexist with low health literacy and high perceived treatment burden in individuals with CVD. The significance of this issue lies in the fact that individuals who are socially disconnected are known to experience a higher incidence of health issues. Furthermore, low levels of health literacy and a higher treatment burden may exacerbate or accelerate these problems. Consequently, the accumulation of risk factors may possibly reflect ‘a perfect storm’, further increasing the risk of adverse outcomes in individuals with CVD. Therefore, it is crucial to address these factors to mitigate negative health outcomes among socially disconnected individuals.

Social (dis)connectedness was largely treated as a personal issue in medical systems until the COVID-19 pandemic [47]. Therefore, knowledge of interventions and solutions countering loneliness and/or social isolation among individuals with CVD is scarce, and practice guidelines for clinicians and other healthcare professionals are even more limited. However, responding to patients’ social needs may be integrated into direct care as part of disease management in primary and secondary care settings [48]. Using the novel Educate, Assess, Respond framework for Addressing Social Isolation and Loneliness, Holt-Lunstand and Perissinotto [47] recommend that clinicians acquire knowledge of and acknowledge the importance of social connections and respond accordingly. This includes recommendations to (1) ‘educate’ patients and healthcare professionals about the importance of social connections to empower patients to take actions to reduce risk, (2) assess social connections periodically in clinical practice, and (3) integrate psychosocial support from care team members and systematically offer referrals tailored to patients’ social needs and preferences as part of clinical treatment. The latter may be achieved by partnering with local community resources [47].

Directing our focus towards effective interventions for addressing social disconnectedness, there is limited knowledge about interventions tailored to individuals with chronic illnesses, such as CVD [48]. Nevertheless, targeted interventions aimed at addressing social disconnection might offer valuable benefits for patients, especially if these interventions exhibit positive effects on health and health-related outcomes while strengthening social connections. A notable example of an intervention addressing health and social connections concurrently is the Group4Health (G4H) intervention [49, 50]. G4H is a structured and manualised intervention designed to heighten awareness of how group memberships impact health. Simultaneously, it assists participants in developing personalised strategies to leverage existing group ties and cultivate new social relations that support connectedness. While the intervention has shown effectiveness in reducing loneliness and improving mental health, G4H has not yet been tested among patients with CVD or other chronic conditions.

In conclusion, actively and systematically addressing social connections in individuals with CVD may yield additional benefits beyond the primary outcomes of reducing loneliness and social isolation. For instance, creating or strengthening social resources may promote health literacy or buffer adverse effects of low basic skills. Social connections may further act as a barrier towards perceiving a high treatment burden or the negative health effects associated with such. Taken together, a need exists for recognising social care as part of patient-centred healthcare [51].

### Limitations

The present study has some limitations despite being based on high-quality data from a representative population-based survey. These include its cross-sectional design, which does not allow for causal inferences. Moreover, data on CVD were self-reported and restricted to only three specific cardiovascular diseases, i.e. acute myocardial infarction, angina pectoris, or stroke. The use of register data from national health registers and a broader set of CVDs, including heart failure and heart valve conditions, would have further strengthened the results. As this study is based on secondary data, only two dimensions of health literacy were included. As such, the full extensive HLQ questionnaire was not included in the health and morbidity survey ‘How are you?’. The study might therefore suffer from construct underrepresentation. Also, the applied threshold value used to identify high treatment burden was based on a purely statistical criterion and had no clinical anchor. As with most survey research, non-response bias cannot be ruled out. However, weights were applied to overcome this bias.

Finally, it is crucial to recognise that social connectedness is a multi-dimensional construct. The degree of an individual’s social connectedness is influenced by various factors, such as the presence of relationships and their respective roles, the tangible or perceived support and inclusion experienced, and the nuanced sense of connection shaped by both positive and negative qualities [52]. However, in the present paper, our attention is directed towards two specific constructs within the domain of social disconnectedness, that is, loneliness and social isolation. Thus, it is important to acknowledge that our exploration does not comprehensively cover all dimensions of social connectedness.

## Conclusion

Loneliness and social isolation were associated with low health literacy reflecting difficulties in ‘understanding health information’ and ‘engaging actively with healthcare providers’. Moreover, loneliness and social isolation were associated with higher odds of a high patient-perceived treatment burden. Thus, the present findings showed that loneliness and social isolation coexist with low health literacy and high treatment burden in individuals with CVD. The implications of these findings for clinical practice are noteworthy as they highlight the need for healthcare providers to address social connections in the context of patient and community care. Furthermore, future research should aim to elucidate the associations between social disconnectedness, health literacy, and treatment burden.
